# The accuracy of Vesical Imaging-Reporting and Data System (VI-RADS): an updated comprehensive multi-institutional, multi-readers systematic review and meta-analysis from diagnostic evidence into future clinical recommendations

**DOI:** 10.1007/s00345-022-03969-6

**Published:** 2022-03-16

**Authors:** Francesco Del Giudice, Rocco Simone Flammia, Martina Pecoraro, Marco Moschini, David D’Andrea, Emanuele Messina, Lucia Martina Pisciotti, Ettore De Berardinis, Alessandro Sciarra, Valeria Panebianco

**Affiliations:** 1 Department of Maternal Infant and Urologic Sciences, “Sapienza” University of Rome, Policlinico Umberto I Hospital, Viale del Policlinico 155, Rome, 00161 Italy; 2 Department of Urology, Stanford Medical Center, Stanford, CA USA; 3 Department of Radiological Sciences, Oncology and Pathology, Policlinico Umberto I Hospital, “Sapienza” University/Policlinico Umberto I of Rome, Viale del Policlinico 155, 00161 Rome, Italy; 4 Department of Urology and Division of Experimental Oncology, Urological Research Institute, Vita-Salute San Raffaele University, Milan, Italy; 5 Department of Urology, Comprehensive Cancer Center, Medical University of Vienna, Vienna General Hospital, Vienna, Austria

**Keywords:** Bladder cancer, Multiparametric magnetic resonance imaging, Muscle-invasive bladder cancer, Bladder cancer staging

## Abstract

**Purpose:**

To determine through a comprehensive systematic review and meta-analysis the cumulative diagnostic performance of vesical imaging-reporting and data system (VIRADS) to predict preoperative muscle-invasiveness among different institutions, readers, and optimal scoring accuracy thresholds.

**Methods:**

PubMed, Cochrane and Embase were searched from inception up to May 2021. Sensitivity (Sn), Specificity (Sp) were first estimated and subsequently pooled using hierarchical summary receiver operating characteristics (HSROC) modeling for both cut-off ≥ 3 and ≥ 4 to predict muscle-invasive bladder cancer (MIBC). Further sensitivity analysis, subgroup analysis and meta-regression were conducted to investigate contribution of moderators to heterogeneity.

**Results:**

In total, *n* = 20 studies from 2019 to 2021 with *n* = 2477 patients by *n* = 53 genitourinary radiologists met the inclusion criteria. Pooled weighted Sn and Sp were 0.87 (95% CI 0.82–0.91) and 0.86 (95% CI 0.80–0.90) for cut-off ≥ 3 while 0.78 (95% CI 0.74–0.81) and 0.94 (95% CI 0.91–0.96) for cut-off ≥ 4. The area under the HSROC curve was 0.93 (95% CI 0.90–0.95) and 0.91 (95% CI 0.88–0.93) for cut-off ≥ 3 and ≥ 4, respectively. Meta-regression analyses showed no influence of clinical characteristics nor cumulative reader’s experience while study design and radiological characteristics were found to influence the estimated outcome.

**Conclusion:**

We demonstrated excellent worldwide diagnostic performance of VI-RADS to determine pre-trans urethral resection of bladder tumor (TURBT) staging. Our findings corroborate wide reliability of VI-RADS accuracy also between different centers with varying experience underling the importance that standardization and reproducibility of VI-RADS may confer to multiparametric magnetic resonance imaging (mpMRI) for preoperative BCa discrimination.

**Supplementary Information:**

The online version contains supplementary material available at 10.1007/s00345-022-03969-6.

## Introduction

The adoption of the Vesical Imaging Reporting and Data System (VI-RADS) criteria based on multiparametric magnetic resonance imaging (mpMRI) of the bladder before trans-urethral resection of bladder tumor (TURBT) has been expanding worldwide [1, 2]. The criteria are established on a standardized 5-point scale assessing the preoperative likelihood of muscle invasiveness**,** but also as potential criterion for predicting final pT stage among bladder cancer (BCa) patients eligible to curative interventions [3]. Despite its relative novel introduction, two diagnostics meta-analysis assessing VI-RADS pooled diagnostic accuracy are already available and showed an excellent promising performance in discriminating non-muscle vs. muscle invasive bladder cancer (NMIBC vs. MIBC) [4, 5]. Still, these preliminary experiences were likely suffering from lack of granularity both in terms of statistical power due to the relatively small number of studies analyzed, and in terms of genito-urinary (GU) readers, MRI scanners and threshold cut-off criteria selected to define MIBC.

VI-RADS is rapidly moving forward into clinical practice. A mounting body of evidence**,** including published prospective data and ongoing clinical trials**,** is indeed exploring the wide flexibility of this novel score for new clinical insights to potentially drive the therapeutic algorithm across different BCa stages. This has so far involved selection criteria for high-risk NMIBC candidate**s** for secondary resection [6], complete/partial radiographic response in MIBC undergoing neoadjuvant regimens [7, 8], as well as those locally advanced cases directly addressed to curative interventions avoiding reliance on extended trans-detrusor resections in favor of adequate biopsy sampling [9].

Despite promising implications of internalizing VI-RADS into therapeutic uro-oncologic algorithm, the following relevant issues still remain unsolved: (I) exploring the optimal diagnostic settings of the score; (II) reproducibility between different MRI scanners and readers with varying experience; (III) defining appropriate threshold cut-off criteria (VI-RADS ≥ 3 vs ≥ 4) for muscle invasiveness-definition. These unmet needs require a definitive statement before further dedicated clinical trials and investigations can be developed to assess predictive value of VIRADS for determining muscle invasion in bladder cancer patients. With this aim we performed an updated comprehensive systematic review of the literature by including all the available international experiences validating the VI-RADS in the pre-TURBT setting for MIBC determination and we provided pooled estimates regarding the diagnostic performance of the score among all available GU readers, MRI scans and VI-RADS cut-off threshold criteria involved.

## Methods

This systematic review and meta-analysis was conducted according to Preferred Reporting Items for Systematic Reviews and Meta-Analyses (PRISMA) guidelines [10]. A research question was established based on the Patient-Index test-Comparator-Outcome-Study design (PICOS) criteria as the following: what is the current cumulative diagnostic performance of VI-RADS scoring criteria for NMIBC vs. MIBC clinical staging discrimination? Furthermore, our goal was to compare current evidence within all available retrospective/prospective and/or single-/multicenter cohort studies applying different MIBC cut-off VI-RADS criteria as compared with histopathological results. In particular, we determined the pooled diagnostic performance estimators among all the available radiologists with different level of expertise and GU-specialized volume institutions.

### Evidence acquisition

We performed a systematic review of the literature in PubMed, Web of Science, Embase, and Cochrane from inception to May 2021, without language restriction, to identify studies that examined the implementation of pre-TURBT VI-RADS scoring criteria for BCa staging purposes and evaluated the pooled diagnostic performance between the radiologists involved. The reference lists of the included studies were also screened for relevant articles. Only original prospective and retrospective cohort studies were included and critically evaluated (Level of Evidence: II and III-a). Case reports, abstracts and meeting reports were excluded from the analysis. Search key terms included with primary and secondary fields have been reported in Supplementary Table 1.

### Selection of the studies and criteria of inclusion

Entry into the analysis was restricted to data collected from original articles that examined patients with primary and/or recurrent BCa diagnosis, which assessed final BCa extension through surgical specimen both from TURBT/Re-TURBT or radical/partial cystectomy (RC), and that aimed to report the standard diagnostic indicators of VI-RADS performance for identifying MIBC preoperatively. Moreover, only those studies including sufficient data to reconstruct 2 × 2 tables with regard to sensitivity and specificity for the outcome of interest (i.e., VI-RADS score cut-off ≥ 3 and cut-off ≥ 4 to predict MIBC) and which assumed “per index lesion” level analysis were considered suitable for further consideration. Additionally, studies were considered eligible if at least one of the involved readers were GU radiologists with at least 5 years’ experience in the GU-MRI imaging, at least one reader was actively involved in the imaging acquisition and revision, and if MRI images acquisition protocol was consistent with what described in the original VI-RADS document [3].

Articles were excluded if they met one or more of the following criteria: inadequate information for data extraction or quality assessment; inclusion of study population consisting of < 15 index lesions; presented outcomes which dealt with other topics (e.g., MRI used to determine clinical T stage (cT) without VI-RADS, or VI-RADS score for each MRI sequence was available but an overall VI-RADS score was not expressed for MIBC detection).

Six authors (FDG, MP, EM, MLP, SF and EDB) independently screened the titles and abstracts of all articles using predefined inclusion criteria. The full-text articles were examined independently by the five (FDG, MP, EM and VP) to determine whether or not they met the inclusion criteria. Final inclusion was determined by consensus of all investigators. Selected articles were then critically analyzed.

The following data were extracted from the included studies by using a standardized form: origin of study (institution and period of enrollment), size of study population, period of time prospectively/retrospectively covered, gold standard for pathological MIBC definition, technical parameters of MRI acquisition (1.5 vs. 3 Tesla magnet, T2WI slice thickness, b values used for DWI, and temporal resolution of DCE-MRI), details regarding MRI interpretation (number of readers, experience of the senior reader, cumulative GU-MRI experience for all the readers involved, and whether they were blinded or not to clinical history), the VI-RADS cutoff value used for determining MIBC on MRI (i.e., cut-off ≥ 3 and cut-off ≥ 4), outcomes related to diagnostic performance of VI-RADS. Finally, baseline clinical and pathological patients and tumor features (e.g., mean/median age, range of patients, number of tumors, percentage of patients with MIBC and histopathological subtypes of tumors screened).

### Assessment of quality for studies included and statistical analysis

To assess the risk of bias (RoB), all included experiences were independently reviewed using the “Quality Assessment of Diagnostic Accuracy Studies” (QUADAS-2) [11], by assessing the potential risk for selection bias, information bias, measurement bias, or confounding bias. Three reviewing authors (FDG, MP and SF) independently assessed the methodological quality based on sequence generation, allocation concealment, blinding of personnel, blinding of outcome assessors, incomplete outcome data, selective outcome reporting, and additional sources of bias. Publication bias was tested both by visual assessment of the Deeks’ funnel plot and calculation of p value using the Deeks’ asymmetry test [13]. We compared diagnostics indicators among the studies at different steps. First, we generated 2 × 2 contingency tables for each GU radiologist involved in each article based on the reported Sn, Sp or raw data and then we used pooled weighted Sn, Sp, likelihood positive and negative ratio (LR + , LR−) with their computed 95% Confidence Intervals (CI) for determining the overall weighted diagnostic estimate for both VI-RADS threshold cut-off ≥ 3 and ≥ 4. Secondly, pooled Sn and Sp estimates were calculated with hierarchical logistic regression modeling, including bivariate and hierarchical summary receiver operating characteristic (HSROC) modeling, and then graphically presented using HSROC curves with 95%CI and prediction regions for both threshold cut-off ≥ 3 and ≥ 4, respectively. Sensitivity analyses were performed to assess the contribution of each study to the pooled estimate by excluding individual trials one at a time and recalculating the pooled estimates for the remaining studies. Evaluation for presence of heterogeneity was done using [12]: (1) Cochran’s *Q* test with *p* < 0.05 signifying heterogeneity; (2) Higgins *I*^2^ test with inconsistency index (*I*^2^) = 0–40%, heterogeneity might not be important; 30–60%, moderate heterogeneity; 50–90%, substantial heterogeneity; and 75–100%, considerable heterogeneity. The pooled weighted Sn, Sp, LR + and LR− estimates were calculated using random effects model. Our results are graphically displayed as forest plots on a per-single GU reader level, with pooled results indicating overall accuracy to discriminate NMI form MIBC using VI-RADS criteria ≥ 3 and ≥ 4, respectively. Furthermore, Fagan nomogram was generated to display the post-test probabilities when the pre-test probability was 25%, which corresponds to the prevalence of MIBC over the years screened.

Subgroup analyses were performed looking at differences in categorical confounders (e.g., Magnet strength [T], MIBC proportion, study design, etc.). Meta-regression analyses were performed using available continuous variables retrieved among the studies. Pooled weighted diagnostic estimates were plotted against the following available quantitative variables: mean/median age of the patients, total number of patients/lesions screened, range of study time screened (months retrospectively or prospectively imputed), the relative percentage of MIBC (% ≥ T2) documented and months from original VI-RADS publication release. Calculations were accomplished using the MIDAS command on Stata version 17.1 (Stata Corporation, College Station, TX, USA).

## Results

### Search results

The initial search yielded *n* = 115 articles (PubMed: 86; Cochrane: 6; and Embase: 23). Forty-four were excluded, as they contained overlapping data or were duplicates appearing in multiple databases. Of the remaining *n* = 71, *n* = 47 were further excluded because did not examine VI-RADS (*n* = 9), contained only MRI-based sequences information (*n* = 5) or were review papers, editorials or abstracts (*n* = 33). Full-text articles were then reevaluated and critically analyzed for the remaining *n* = 24 journal references. Within this in-depth review, a further *n* = 4 did not meet the inclusion criteria. The remaining *n* = 20 studies were included in our review (Supplementary Fig. 1). No study was considered to be seriously flawed as per the “Quality Assessment of Diagnostic Accuracy Studies”. Studies’ risk to performance bias was overall low with some attrition bias due to incomplete outcome data across all the studies. Individual RoB as well as visual assessment of the Deeks’ funnel plots are illustrated in Supplementary Table 2 and Fig. 3, respectively.

### Location, design, and characteristics of the studies population

Patient, tumor, and study characteristics are summarized in Table 1. Of the 20 included articles, *n* = 11 were conducted in Asia (*n* = 5 in China [15–17], *n* = 4 in Japan [18–21], *n* = 2 in Korea [22, 23]), *n* = 6 in Europe (*n* = 4 in Italy [6, 24–26], *n* = 1 in Spain [27]) *n* = 1 in Turkey [28] and 3 in other continents (2 in Egypt [29, 30], 1 in Brazil [31]). Range of eligible study time was comprised in between 2019 and 2021 and included patients who had been treated for BCa between 2005 and 2020. All eligible articles were single centered with Metwally et al. [30] representing the only available multicenter experience. Out of these, *n* = 14 were retrospective and *n* = 6 were prospective with cumulative sample size ranging from 18 up to 340 patients (Table 1).

**Table 1:** Clinical and demographic characteristics of the studies enrolled in the systematic review and meta-analysis

Study	Year of publication	Study design	Centers	Time (month)	Age (years)	Patients (*n*)	Lesions (*n*)	T stage	MIBC (%)	UCUB (%)	From submission (mo)	From acceptance (mo)
Barchetti et al	2019	Retrospective	Single	10	69	75	103	Ta-T3a	29%	100%	20	19
Ueno et al	2019	Retrospective	Single	92	73	74	74	≥ Tis	50%	96%	NA	26
Wang H et al	2019	Retrospective	Single	80	64	340	340	Ta-T4	25%	100%	30	26
Makboul et al	2019	Prospective	Single	NA	57	50	50	T1-T4	36%	NA	24	19
Del Giudice et al	2019	Prospective	Single	18	67	231	231	Ta-T4	27%	100%	NA	19
Kim SH	2019	Retrospective	Single	38	66	297	339	Tis-T4	30%	94%	NA	22
Hong et al	2020	Retrospective	Single	6	73	32	66	NA	25%	NA	20	14
Marchioni et al	2020	Prospective	Single	NA	73	68	68	Ta- T3	10%	NA	14	14
Sakamoto et al	2020	Retrospective	Single	67	73	176	176	≥ Ta	26%	100%	17	13
Liu et al	2020	Retrospective	Single	17	68	126	126	Ta-T4	39%	100%	19	14
Vaz et al	2020	Retrospective	Single	35	68	30	30	NA	27%	93%	24	22
Wang Z et al	2020	Retrospective	Single	29	66	220	220	Ta-T4	51%	NA	19	13
Arita et al	2020	Retrospective	Single	94	74	66	66	Ta-T3	26%	100%	13	8
Etxano et al	2020	Retrospective	Single	4	70	18	18	Tis-T2	33%	99%	14	6
Ueno et al	2020	Retrospective	Single	96	73	91	91	Ta-T2	53%	100%	12	9
Metwally et al	2021	Prospective	Multi	18	62	331	331	NA	57%	100%	5	3
Akcay et al	2021	Prospective	Single	22	68	73	73	NA	42%	100%	16	2
Meng et al	2021	Retrospective	Single	7	60	61	89	Ta-T4	31%	NA	4	7
Delli Pizzi et al	2021	Prospective	Single	12	73	38	38	Ta-T3	18%	100%	9	7
Li et al	2021	Retrospective	Single	16	61	80	80	Ta-T4	34%	100%	2	1

*MIBC* muscle-invasive bladder cancer; *UCUB* urothelial carcinoma of the urinary bladder; *NA* not applicable

Across eligible studies, median age was between 57 and 74 years old. Four studies analyzed independently more than one lesion per patients [16, 22–25] and consequently the total amount of lesions investigated exceeds the number of patients included (2609 vs 2477). Nevertheless**,** all these studies reported VI-RADS accuracy relying on the index lesions identified. All the studies relied on histopathological report from TURBT and/or partial cystectomy and/or RC, performed within 1 to 12 weeks from MRI. MIBC rates retrieved among the experiences included ranged from 10 to 53%. Moreover, the majority of the studies included only urothelial bladder cancer, with other variant histology accounting only for **a** relative limited proportion in each study (from 1 to 6%) (Table 1).

### Technical imaging modalities and reader characteristics

The MRI parameters and GU reader characteristics of each study are summarized in Table 2. For imaging acquisition protocols, *n* = 12 studies [6, 13–17, 20, 22–26] relied on 3 T scanners, *n* = 6 [21, 27–31] on 1.5 T and the remaining *n* = 2 [18, 19] on either 1.5 or 3 T scanners, with T2WI reported slice thickness ranging from 2 to 5 mm. In all the enrolled studies, MRI findings were always interpreted blinded to the clinical and pathological patient’s history. Of note, regardless form classic VI-RADS accuracy and reproducibility trials, *n* = 5 experiences focused on potential approaches to improve VI-RADS score diagnostic performance. Specifically, Akcay et al. [28] analyzed tumor contact length as a parameter to improve the accuracy of VI-RADS score 3. Moreover, Li et al. and Sakamoto et al. [17, 20] implemented VI-RADS with standardized tumor apparent diffusion coefficients (st-ADC) and volumetric ADC histogram analysis, respectively. Additionally, Arita et al. [21] and Meng et al. [16] relied on 3D fast spin echo (FSE) T2-weighted acquisition, instead of classical 2D FSE T2-weight acquisition and bi-planar reduced field-of-view DWI (rFOV DWI). Finally, Delli Pizzi et al. [26] compared VIRADS score relying on contrast-free bi-parametric vs multiparametric MRI. Among the aforementioned studies, our analysis was, however, focused on the standard VI-RADS outcomes reported and relied on the reporting findings dichotomized for cut-off score ≥ 3 vs. ≥ 4.

**Table 2:** Radiological and MRI-specific characteristics of the studies enrolled in the systematic review and meta-analysis

Study	Year	Readers (*n*)	1st reader experience (years)	Cum readers’ experience (years)	Magnetic field (*T*)	T2WI slice thickness (mm)	DWI b (s/mm^2^)	DCE temporal resolution (s)	Interval MRI-surgery (weeks)	Ref. for diagnosis
Barchetti et al	2019	2	10	15	3	3–4	0–88–1000–2000	5″	< 6	TURBT
Ueno et al	2019	5	> 20	NA	1.5/3	4	0–1000	40″− 80″− 120″− 160″	NA	TURBT
Wang H et al	2019	2	32	40	3	NA	NA	(20″− 131″) × 5	< 2	TURBT/Cystectomy
Makboul et al	2019	2	NA	NA	1.5	3	0–400–800–1000	20″− 70″− 180″	NA	TURBT
Del Giudice et al	2019	2	25	37	3	3–4	0–800–1000–2000	5″	< 6	TURBT/Cystectomy
Kim SH	2019	2	12	18	3	3	0–1000	30″ x (4–6)	NA	TURBT/Cystectomy
Hong et al	2020	3	24	47	3	4	0–50–800–1000	30″	2	TURBT/Cystectomy
Marchioni et al	2020	2	> 20	NA	3	3–4	0–600-1000–1500–2000	30″	NA	TURBT
Sakamoto et al	2020	2	15	24	3	4–5/3.5	0–1000/0–2000	NA	NA	TURBT
Liu et al	2020	2	NA	NA	3	4–5	0–50-800–1000	(20″− 131″) × 5	2	TURBT/Cystectomy
Vaz et al	2020	2	13	NA	1.5	NA	50–400–800	60″− 300″	1–2	TURBT
Wang Z et al	2020	2	NA	NA	3	NA	NA	NA	2	TURBT/Cystectomy
Arita et al	2020	2	18	30	1.5	2	0–1000	40″	2	TURBT/Cystectomy
Etxano et al	2020	2	4	8	1.5	3	0–800–1000	7″	NA	TURBT
Ueno et al	2020	7	> 20	NA	1.5/3	4	0–1000	30″− 60″− 90″− 120″− 180″	12	TURBT/Cystectomy
Metwally et al	2021	4	16	41	1.5	4	0–800–1000	5″	1	TURBT
Akcay et al	2021	2	20	25	1.5	3	0–1800	8″	NA	TURB/Cystectomy
Meng et al	2021	2	10	18	3	4	0–800	NA	2	TURBT
Delli Pizzi et al	2021	4	15	31	3	4	600–1000–1500–2000	NA	NA	TURBT
Li et al	2021	2	16	24	3	4	0–800	NA	NA	TURBT/Cystectomy

*T2WI* T2-weighted imaging; *DWI* diffusion-weighted imaging; *DCE* dynamic contrast enhanced; *NA* not applicable

According to single reader diagnostic performance availability, a total of 53 GU radiologists ranging from two up to seven for each study was identified. Only five studies included more than 2 readers [18, 19, 23, 26, 30]. Moreover, two studies included residents among the eligible readers [26, 31] and one reported inexperienced radiologist [19]. However, only *n* = 7 [16, 18, 19, 21, 24, 26, 28] of the eligible studies reported diagnostic performance for all included readers [16, 19, 21, 24, 26, 28]. The overall cumulative level of radiologist experience was declared in *n* = 13 studies [6, 13, 16, 17, 20–24, 26–28, 30]. It varied from a cumulative GU experience per study of 8 up to 47 years and from a single radiologist expertise varying from 4 up to 20 years. Finally, regarding the VI-RADS score thresholds adopted, n = 12 studies reported extractable data on both VI-RADS ≥ 3 and VI-RADS ≥ 4**,** as cut-off defining the probability of MIBC before staging resection**. **Conversely, *n* = 6 and *n* = 2 studies reported only cut-off ≥ 3 or ≥ 4, respectively. Out of these, only five studies provided raw data for estimating diagnostic performance according to different cut-off.

### Diagnostic performance of VI-RADS cut-off ≥ 3 for MIBC detection

**Fig. 1:**
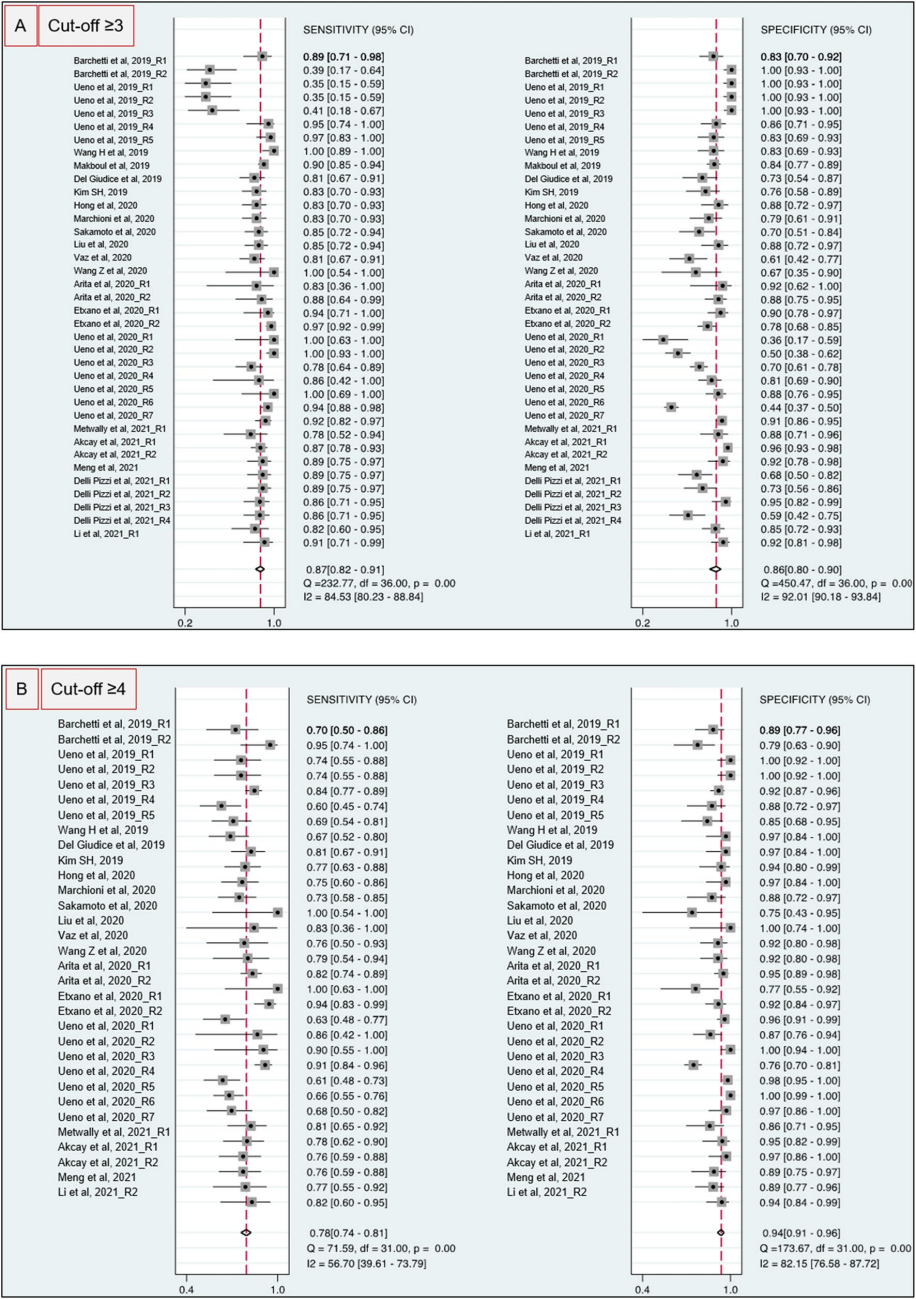
Forest plot of pooled sensitivity and specificity for VIRADS ≥ 3 (**A**) or ≥ 4 (**B**) as the cut-off criterion for MIBC identification

**Fig. 2:**
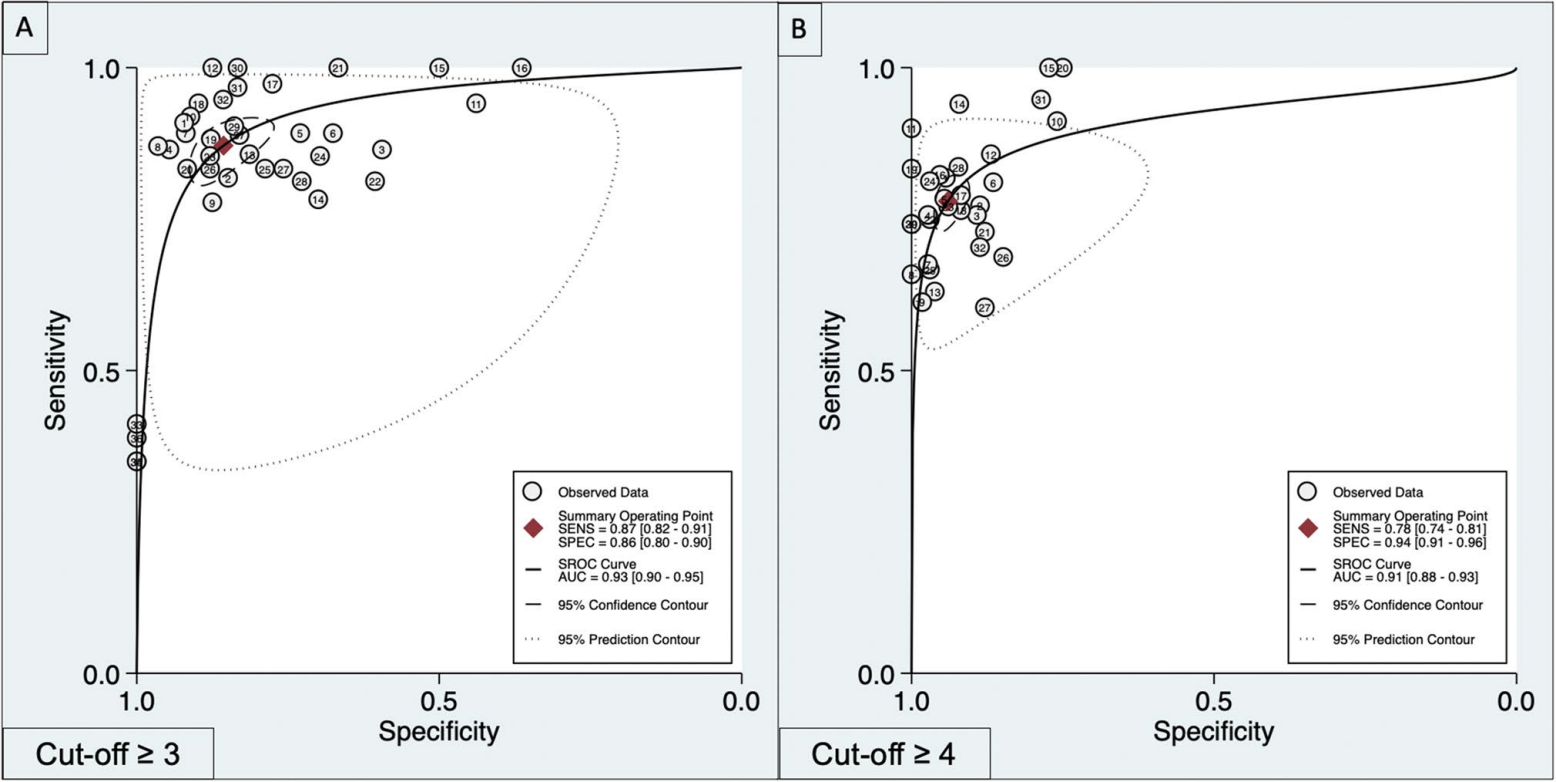
HSROC for diagnostic performance of studies using VI-RADS predicting MIBC. *HSROC* hierarchical summary receiver operating characteristics; *VI-RADS* vesical imaging reporting and data system

**Fig. 3:**
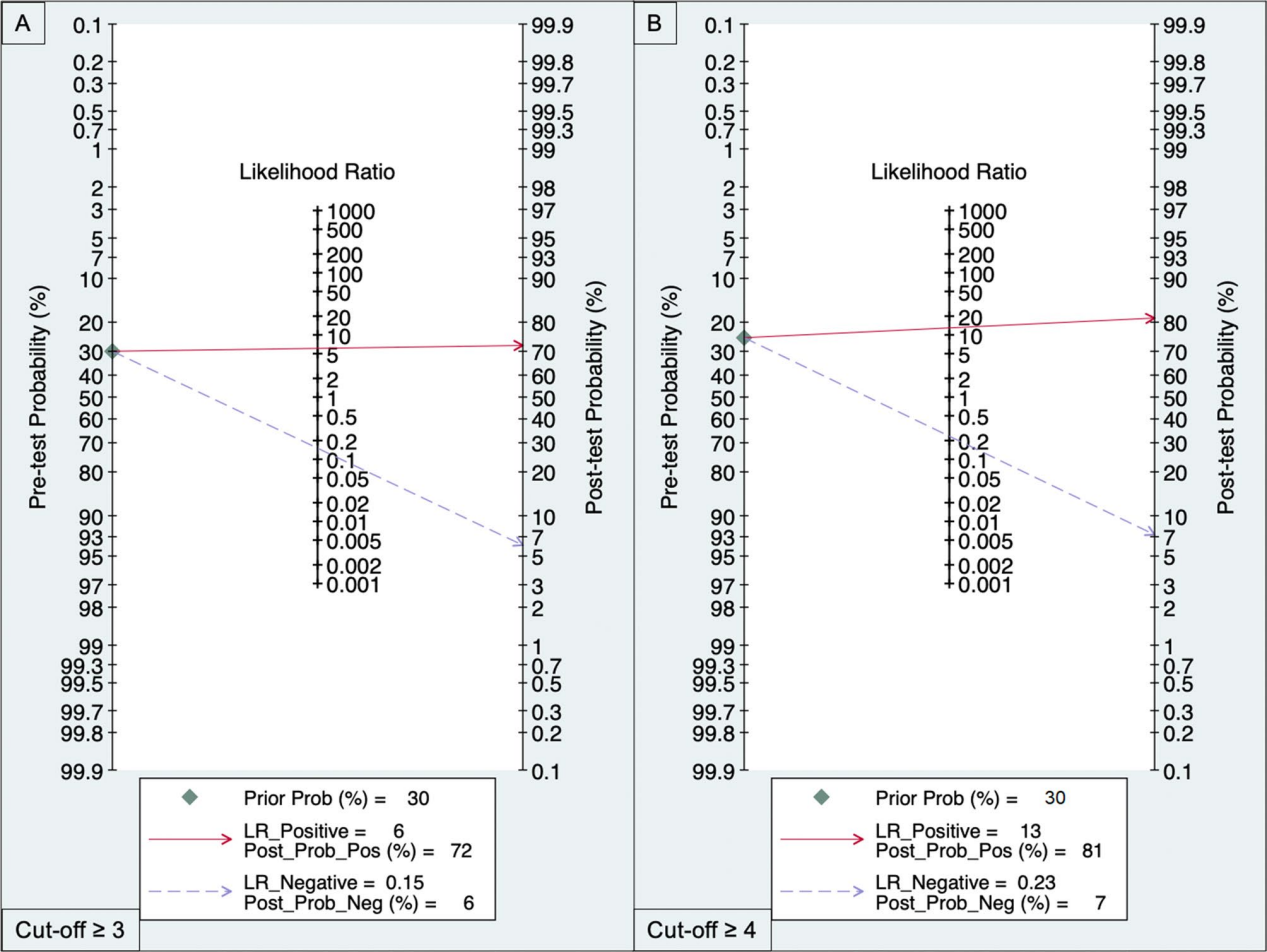
Fagan nomogram reflecting pre- and post-test probability estimation for clinical utility of VIRADS criterion ≥ 3 (**A**) or ≥ 4 (**B**), respectively. *VI-RADS* vesical imaging reporting and data system

All the 20 [13–31] studies enrolled reported diagnostic accuracy information for VI-RADS cut-off ≥ 3 for MIBC. Pooled paired Sn and Sp were 0.87 (95% CI 0.82–0.91) and 0.86 (95% CI 0.80–0.90) (Fig. 1A), while pooled LR + and LR− were 6.1 (95% CI 4.3–8.5) and 0.15 (95% CI 0.11–0.20), respectively. The area under the HSROC curve was 0.93 (95% CI 0.90–0.95; Fig. 2A). Given a pre-test probability of 30%, Fagan nomogram showed a positive and negative post-test probability for detecting MIBC of 72% and 6%, respectively (Fig. 3A). As there was evidence for the presence of substantial heterogeneity within the studies, the analyses reported results according to a random-effect model. Inspection of funnel plot suggested that for all the 20 studies together, there was no significant small-study effect with no study tending to have a higher outliner estimate, as depicted in Supplementary Fig. 3. Indeed, Egger’s regression test showed non-significant small-study effect (*p* = 0.58) whilst the ‘Trim and Fill’ method suggested that no ‘missing’ studies would need to be included to remove asymmetry from the funnel plots. Furthermore, at sub-group analysis, the study design, as well as the magnetic strength and slice thickness adopted revealed to be the source for major heterogeneity among the studies. Meta-regression and sub-analysis plots have been summarized in Supplementary Fig. 4A.

### Diagnostic performance of VI-RADS cut-off ≥ 4 for MIBC detection

*N* = 18 [6, 13, 14, 16–25, 27, 28, 30] out of 20 studies reported or had extractable information regarding VI-RADS cut-off criteria ≥ 4. Weighted pooled Sn and Sp were 0.78 (95% CI 0.74–0.81) and 0.94 (95% CI 0.91–0.96) (Fig. 1B), while pooled LR + and LR− were 13 (95% CI 9.2–18.2) and 0.23 (95%CI: 0.20 – 0.27), respectively. The area under the HSROC curve was 0.91 (95% CI 0.88–0.93; Fig. 1B). Given a pre-test probability of 30%, Fagan nomogram showed a positive and negative post-test probability for detecting MIBC of 81% and 7%, respectively (Fig. 2B**)**. Similarly, to cut-off ≥ 3, the inspection of Funnel’s plot revealed absence of asymmetry with no significant small-study effect as depicted in Supplementary Fig. 3. Additionally, Egger’s regression test confirmed a non-significant small-study effect (*p* = 0.26) whilst the ‘Trim and Fill’ method reveled that no ‘missing’ studies would need to be included to remove asymmetry from the funnel plots. Furthermore, the source for heterogeneity was confirmed to be dependent mainly form the study design, together with the use of different magnetic strength and slice thickness as depicted in Supplementary Fig. 4B.

## Discussion

The first diagnostic meta-analysis by Woo et al. [4] assessing *n* = 6 studies with a total of 1170 patients**,** who underwent mpMRI of the pelvis before TURBT, proved relevant enthusiasm toward the urologic academic community for the VI-RADS scoring criteria and its diagnostic ability to preoperatively provide reliable clinical BCa staging. The article was indeed released after less than 24 months from the original VI-RADS document [3] and reported a pooled sensitivity and specificity for predicting MIBC of 0.83 (95% CI 0.70–0.90) and 0.90 (95% CI 0.83–0.95) while the area under the HSROC curve was 0.94 (95% CI 0.91–0.95). Similarly, Luo et al. [5] in a separate and independent meta-analysis obtained an overlapping area under the HSROC curve of 0.93 (95% CI 0.91–0.95). Following these preliminary but enthusiastic findings, van der Heijden and Witjes [32] have reasonably and rightly addressed the lack of data regarding reproducibility of the scoring system among different centers, linked to both radiologists’ varying expertise and the MRI scanners (e.g., magnetic field, vendor etc.), thus warranting the need for lager updated analysis and cautiously advocating the need for more data and experience before providing definitive statement, especially for the potential inclusion of VI-RADS in the diagnostic algorithm of BCa.

These considerations appear now particular timely given a complementary growing body of evidence is emerging**,** exploring not solely the diagnostic performance indexes of VI-RADS itself, but also the potential novel implications**,** which could derive from the internalization of a reliable preoperative staging tool in the decision-making process of daily urological practice. For example, Del Giudice et al. [6] have reliably demonstrated the ability of VI-RADS score 2 as a clinical predictor of non-invasive disease at Re-TURBT in those high-risk NMIBCs candidate for eventually avoiding early repeated resection. At the same time VI-RADS cut-off ≥ 3 was considered as for predicting under-staged MIBC at TURBT with the intent for future identification of those false-negative cases that should definitely not miss Re-TURBT.

Finally, the most ambitious available but still ongoing Bladder Path Trial (www.birmingham.ac.uk/bladderpath) is testing whether TURBT can be substituted by mpMRI**,** after bladder biopsy has proven the presence of bladder cancer**,** for determining if the patient will be submitted to conservative rather radical interventions.

All these emerging landscapes have led us to develop an updated comprehensive meta-analysis pooling the cumulative diagnostic performance of both VI-RADS cut-off criterions for MIBC discrimination and furthermore to investigate the contribution of clinical and radiological confounders to the diagnostic accuracy with the aim to provide useful recommendations for future trials and investigations.

The first reassuring finding from our analysis was that VI-RADS score confirmed its excellent performance regardless of the cut-off adopted (HROC: 0.93, 95% CI 0.90–0.95 and 0.91, 95% CI 0.88–0.93, respectively; *p* = 0.16). Nevertheless, the implementation of one threshold criterion over another demonstrated specific diagnostic applicability**,** which could be adapted and implemented in different clinical spectrum. In particular, MIBC defined by VI-RADS cut-off ≥ 4 was clearly associated with greater specificity and LR + (0.94, 95% CI 0.91—0.96 and 13, 95% CI 9.2—18.2 respectively) reaching a post-test positive probability of 81% compared to 72% for cut-off ≥ 3. Such difference should therefore translate into recommendations for future research were VI-RADS would be utilized to address patients directly into invasive and radical interventions**,** such as NAC ± RC**,** as for example proposed in the Bladder Path trial. In line with these findings, recently, Del Giudice et al. [9] reported VI-RADS score 5 having the highest ever reported diagnostic performance in identifying locally advanced BCa with extravesical involvement (sensitivity 90.2%; specificity 98.1%; AUC 94.2%, 95% CI 88.7–99.7%), thus delineating an even more reliable imaging-based risk profile of patients**,** who could avoid the reliance and morbidity of invasive diagnostic TUR, and therefore could receive definitive treatments without time-consuming sequalae related to the staging procedures. On the contrary, VI-RADS cut-off ≥ 3, given its broader inclusivity of even those VI-RADS score 3 suspicious lesions, delineates the shape of a predictive tool with better sensitivity and LR − , which would decrease the misdiagnosis of MIBC. For these properties, the utilization of such criterion should be preferred in those trials evaluating patients undergoing pathologic confirmation of muscle invasiveness (e.g., selection of high-risk NMIBCs candidate for Re-TURBT). All these considerations find an appropriate support from the maturity and robustness of the data presented in our analysis. We were indeed able to compare the highest ever reported number of experiences validating VI-RADS for MIBC diagnosis in the pre-TURBT setting. More interestingly, our analysis was based on a per-single reader contribution**,** thus expanding the heterogeneity of different cumulative level of GU radiological expertise worldwide and therefore closely mirroring daily VI-RADS clinical reproducibility.

Our study is, however, not devoid of limitations. First and more importantly, we would readily acknowledge the existence of variable heterogeneity and the risk of bias deemed by the quality of the included studies which exhibit some differences in terms of study designs (retrospective, prospective, single institution, multi-center, etc.), magnetic strength and MR imaging characteristics. Nevertheless, we deeply investigated through sensitivity, subgroup and meta-regression analyses all the possible available confounders on a per single-reader level in order to balance and model the contribution of clinical and radiological variables to the overall effect size. Of note, differently form the two previous existing meta-analysis, the sample size enrolled in each study was resized as non-significant confounder. Conversely, we confirmed a trend toward greater diagnostic performance in those studies adopting 3 T MR magnet with thinner slices (3 mm).

Finally, according to our updated findings together with the already available data regarding high GU inter-reader variability [33], the shape of this innovative and versatile imaging tool seems confirmed, with potential useful indications which may be further internalized in upcoming clinical trials.

## Conclusion

VI-RADS is a diagnostic tool characterized by excellent diagnostic performance regardless its score cut-off criterion for defining MIBC in the pre-TURBT setting. Our analysis further supports the adoption of VI-RADS ≥ 3 or ≥ 4 in certain clinical settings, spanning from accurate diagnostic staging purpose to patient-level therapeutic algorithm personalization.

## Supplementary Information

Below is the link to the electronic supplementary material.

Supplementary file1 PRISMA flow diagram (PNG 312 KB)

Supplementary file2 Risk of bias assessment according to quality assessment of diagnostic accuracy studies (QUADAS-2). *RoB* risk of bias; +: low risk of bias; ?: unclear risk of bias; -: high-risk of bias (PNG 2596 KB)

Supplementary file3 Deeks’ funnel plot for assessment of publication bias among both VI-RADS criterion ≥ 3 (**A**) or ≥ 4 (**B**). *ESS* effective sample size (PNG 613 KB)

Supplementary file4 Univariable meta-regression and sub-group analysis for both studies assessing VIRADS criterion ≥3 (**A**) or ≥4 (**B**) respectively. *BCa* bladder cancer; *MIBC* muscle-invasive bladder cancer. *T* tesla (PNG 753 KB)

Supplementary file5 Comprehensive list of search terms for primary and secondary fields (DOCX 14 KB)
