# Supplementary figures and images for: The accuracy of Vesical Imaging-Reporting and Data System (VI-RADS): an updated comprehensive multi-institutional, multi-readers systematic review and meta-analysis from diagnostic evidence into future clinical recommendations
Source: World J Urol. 2022 Mar 16;40(7):1617–28. doi: 10.1007/s00345-022-03969-6 (PMC9237003; doi:10.1007/s00345-022-03969-6)

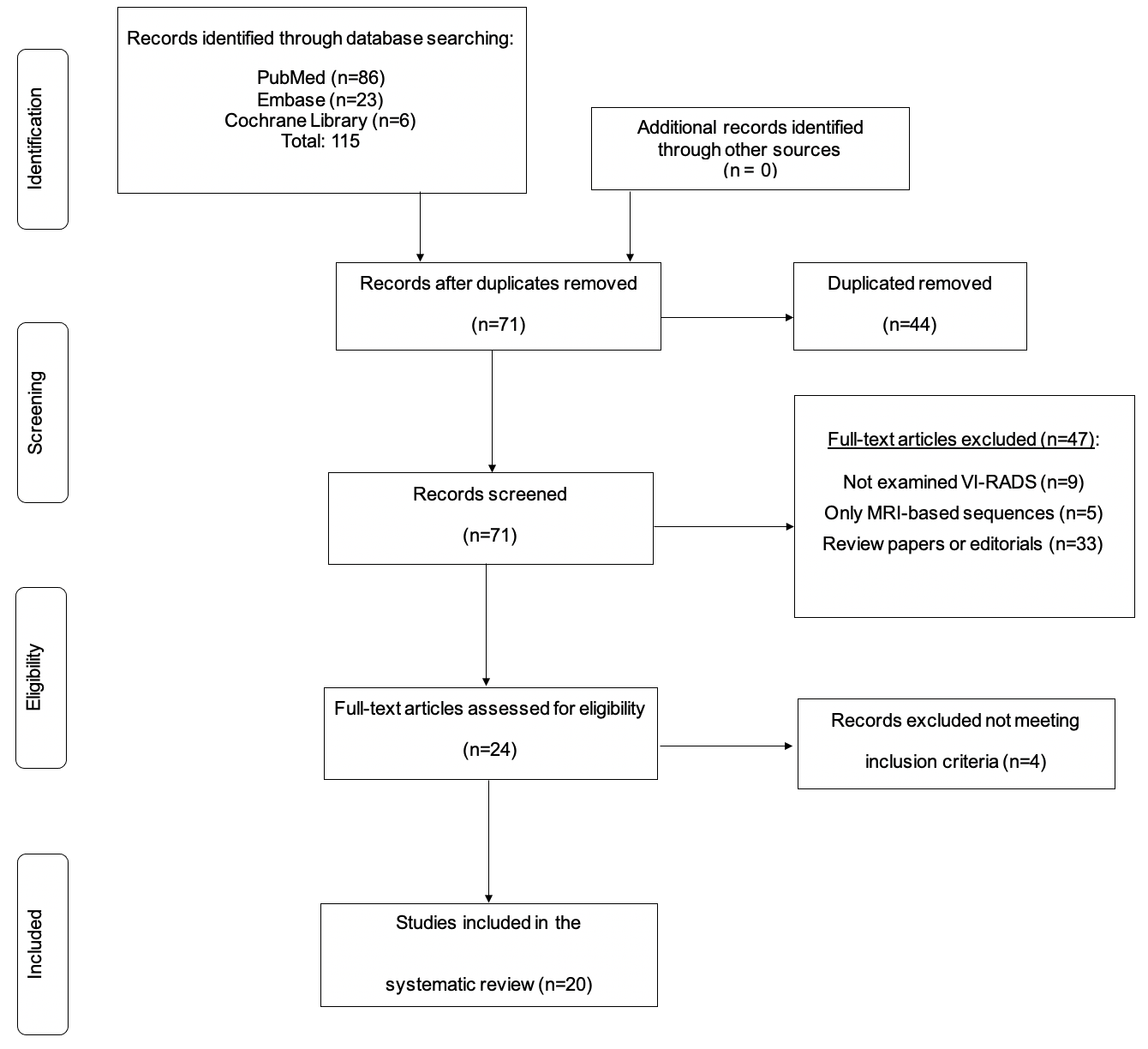

Supplement: Supplementary file 1 — Supplementary file1 PRISMA flow diagram (PNG 312 KB) [file 345_2022_3969_MOESM1_ESM.png]

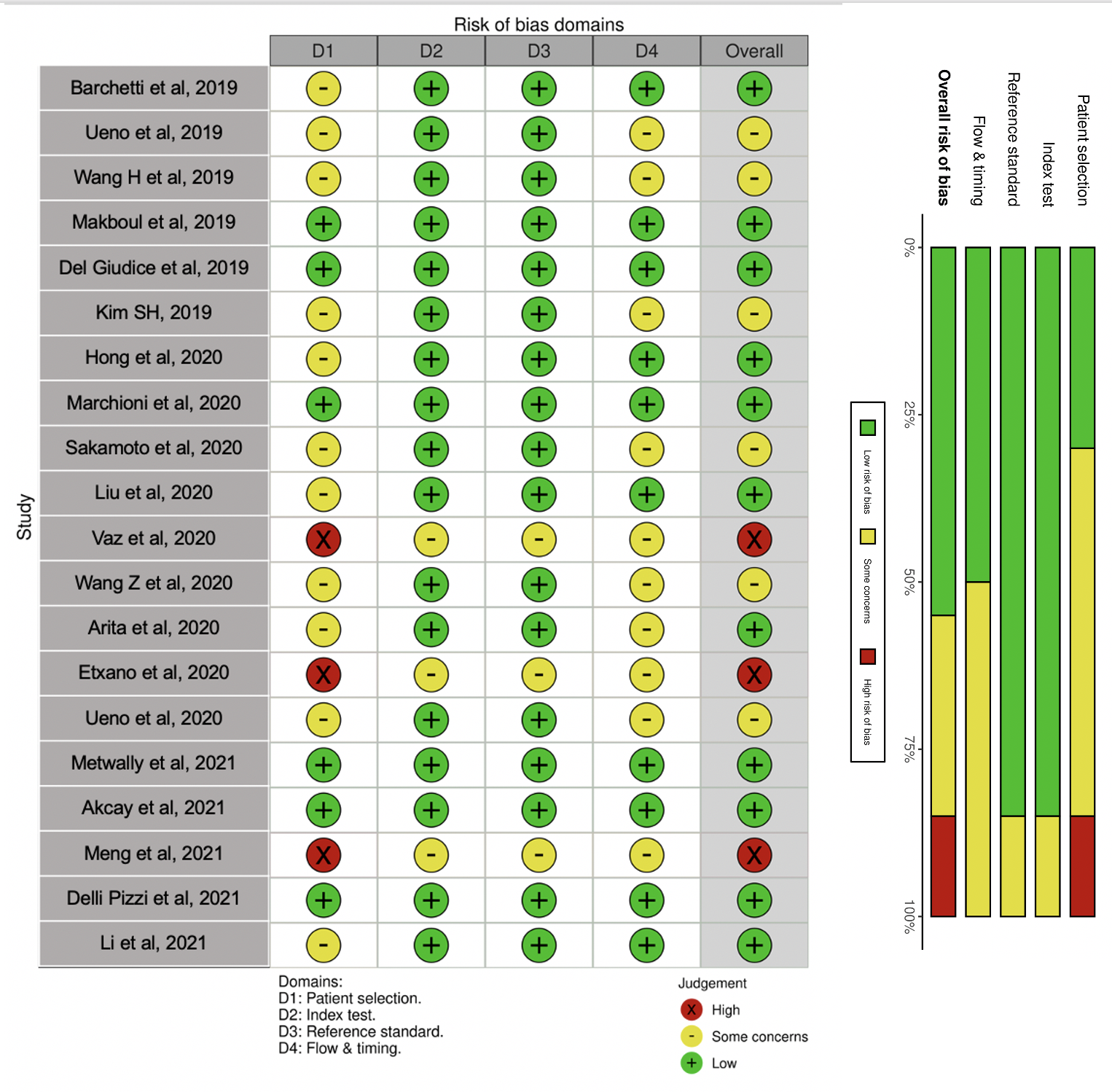

Supplement: Supplementary file 2 — Supplementary file2 Risk of bias assessment according to quality assessment of diagnostic accuracy studies (QUADAS-2). RoB risk of bias; +: low risk of bias; ?: unclear risk of bias; -: high-risk of bias (PNG 2596 KB) [file 345_2022_3969_MOESM2_ESM.png]

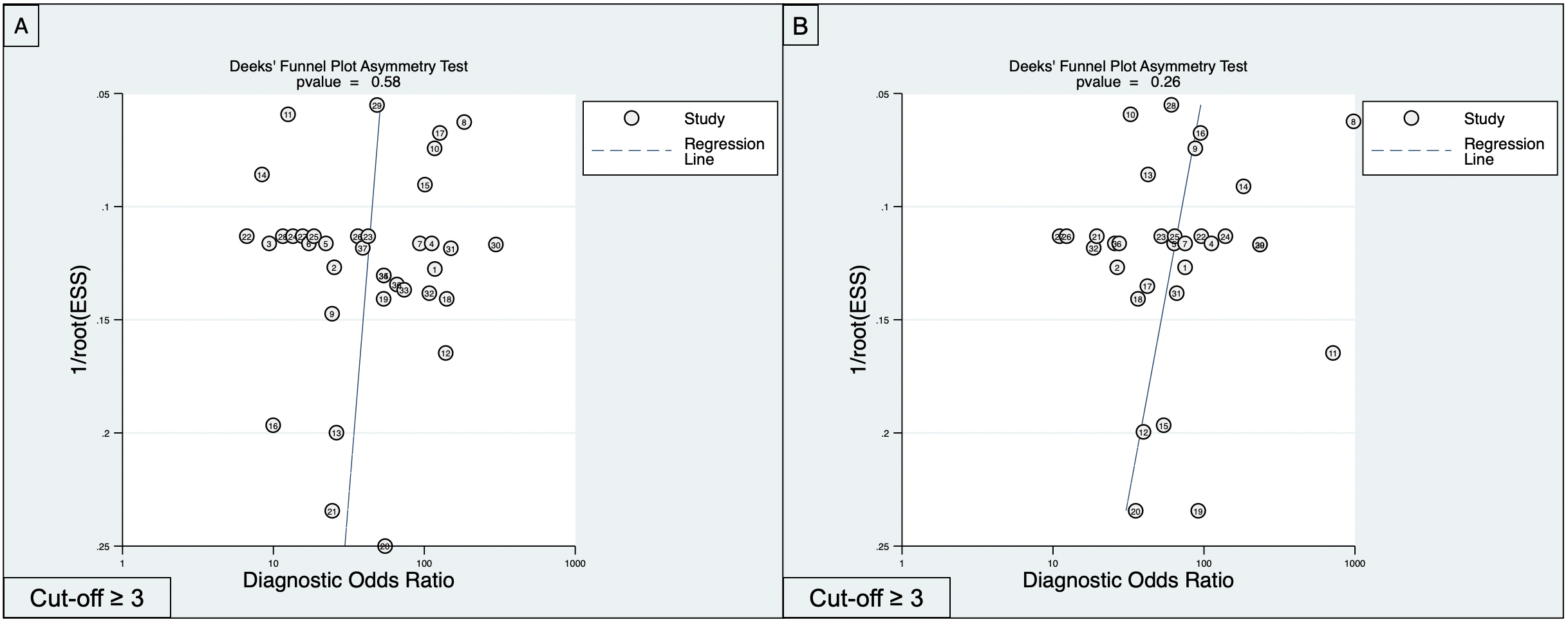

Supplement: Supplementary file 3 — Supplementary file3 Deeks’ funnel plot for assessment of publication bias among both VI-RADS criterion ≥ 3 (A) or ≥ 4 (B). ESS effective sample size (PNG 613 KB) [file 345_2022_3969_MOESM3_ESM.png]

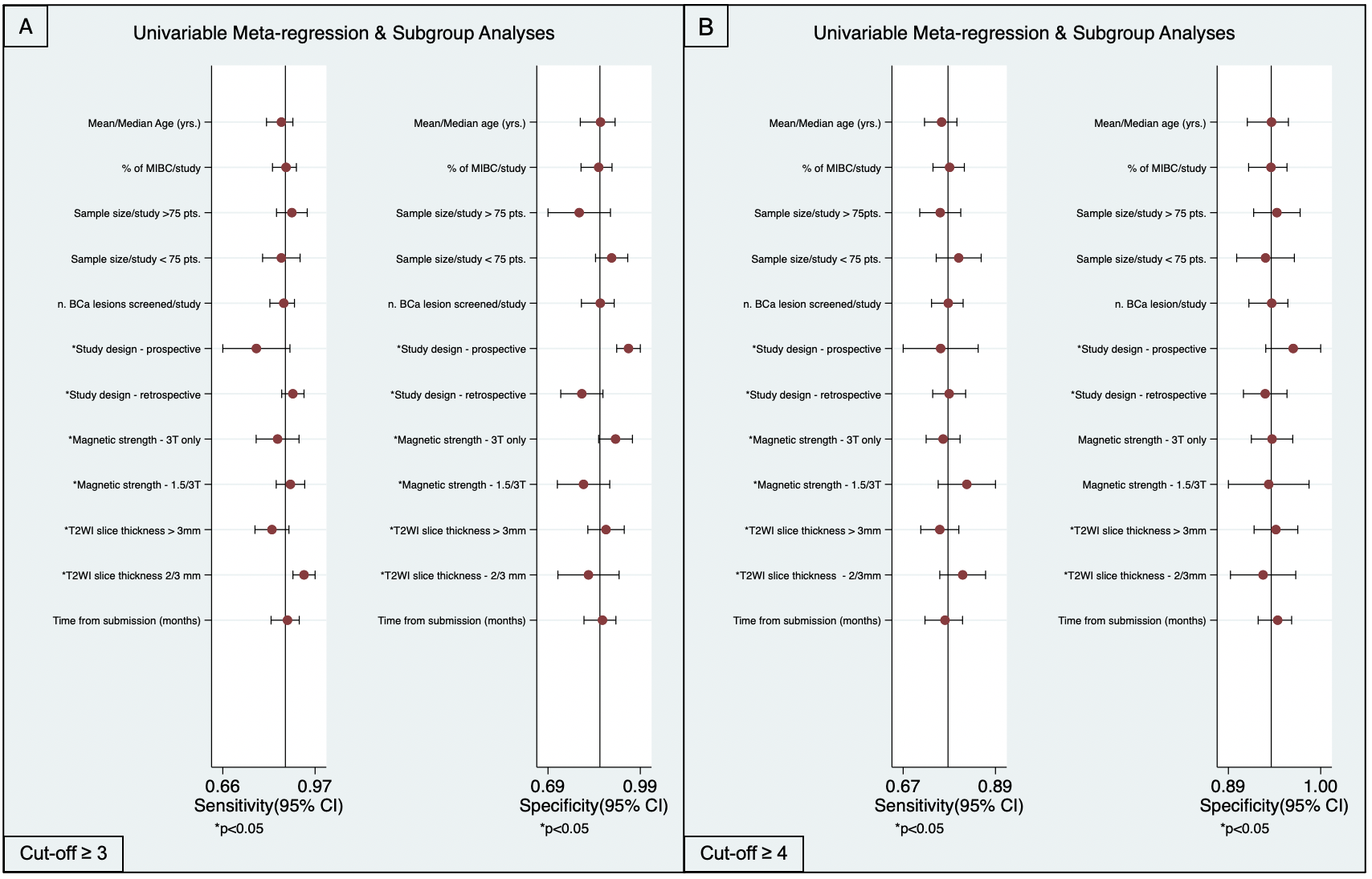

Supplement: Supplementary file 4 — Supplementary file4 Univariable meta-regression and sub-group analysis for both studies assessing VIRADS criterion ≥3 (A) or ≥4 (B) respectively. BCa bladder cancer; MIBC muscle-invasive bladder cancer. T tesla (PNG 753 KB) [file 345_2022_3969_MOESM4_ESM.png]
