# Supplementary material for: The accuracy of Vesical Imaging-Reporting and Data System (VI-RADS): an updated comprehensive multi-institutional, multi-readers systematic review and meta-analysis from diagnostic evidence into future clinical recommendations
Source: World J Urol. 2022 Mar 16;40(7):1617–28. doi: 10.1007/s00345-022-03969-6 (PMC9237003; doi:10.1007/s00345-022-03969-6)
Supplement: Supplementary file 5 — Supplementary file5 Comprehensive list of search terms for primary and secondary fields (DOCX 14 KB) [file 345_2022_3969_MOESM5_ESM.docx]

**Supplementary Table 1.** Comprehensive list of search terms for primary and secondary fields.

| Key words (primary field) | Key words (secondary field) |
| --- | --- |
| Bladder Cancer | Non-Muscle Invasive Bladder Cancer and Magnetic Resonance Imaging |
| Vesical Imaging-Reporting and Data System | Muscle-Invasive Bladder Cancer and Magnetic Resonance Imaging |
| VI-RADS | VI-RADS sensitivity |
| VIRADS | VI-RADS specificity |
| Multiparametric Magnetic Resonance Imaging | VI-RADS area under the curve |
| mpMRI | Bladder cancer stage discrimination |
| Diagnostic accuracy | Prospective Cohort Studies |
| Diagnostic performance | Retrospective Cohort Studies |
| Bladder Cancer Clinical Staging | Year of publication |
| Bladder Cancer Diagnosis | Genitourinary Radiologist |
